# Mapping the Effectiveness of Compensatory Stepping Training for Balance and Fall Prevention in Community-Dwelling Older Adults: A Scoping Review

**DOI:** 10.7759/cureus.108685

**Published:** 2026-05-11

**Authors:** Radha R Bhattad, Sudhir L Tungikar

**Affiliations:** 1 Physiotherapy, Pravara Institute of Medical Sciences, Loni, IND; 2 Physiotherapy, Sancheti Institute for Orthopedics and Rehabilitation College of Physiotherapy, Pune, IND; 3 Medicine, Dr. Balasaheb Vikhe Patil Rural Medical College, Loni, IND

**Keywords:** balance, compensatory stepping training, fall, older adults, perturbation

## Abstract

Among older adults, falls are a leading cause of injury, mortality, and hospitalization globally. For fall prevention, perturbation-based protocols targeting reactive responses are used in compensatory stepping training (CST). However, compared with conventional interventions, evidence on the efficacy of CST remains underexplored. Hence, this scoping review maps the current evidence on the effectiveness and outcomes of CST in older adults for reducing falls and enhancing balance confidence compared with conventional interventions, thereby identifying gaps and providing recommendations. A comprehensive literature search was conducted in the PubMed and ScienceDirect databases regarding CST versus conventional therapy from January 2000 to December 2025. Seven studies (n = 12-102; 2008-2021) were included based on the eligibility criteria. Conventional therapies showed comparable functional gains but limited perturbation transfer. CST improved reactive balance, with 31% fewer recovery steps, 15-17% faster step initiation, and improved kinematic adaptability, along with 56-74% prospective fall reductions and high feasibility, reflected by 75-100% adherence and no adverse events. Frail subgroup exclusion, small sample sizes, and short follow-up periods were identified as gaps. For fall prevention and reactive balance, CST offers task-specific promise in terms of kinematic efficiency and scalability. To address psychological outcomes and optimize clinical integration, future trials should focus on multidirectional protocols with larger samples, including high-risk populations, along with assessment of long-term effects.

## Introduction and background

Globally, falls are a leading cause of injury, mortality, and hospitalization and serve as a major public health concern among older adults, encompassing both healthy community-dwelling individuals and those with pathologies or frailty [1-6]. To mitigate falls and associated injuries, general exercise programs, which are evidence-based interventions consisting of combinations of balance, aerobic, and strength training, are necessary for reducing healthcare burdens and enhancing quality of life [7,8]. However, because of limited integration of task-specific strategies and insufficient focus on postural adjustments during unexpected balance loss, evidence remains inconsistent regarding their efficacy in high-risk or frail older populations, in whom fall rates persist at elevated levels [9-13].

The ability to respond adeptly to a balance perturbation, or a “loss of balance,” determines whether it progresses into a fall. Volitional movements such as turning, bending, and reaching, as well as slips, trips, and collisions, often lead to such perturbations. Hence, for effective fall prevention, a targeted focus on balance-recovery reactions is required. For stabilization against varying magnitudes of perturbations, change-in-support balance reactions are pivotal and serve as the primary defense. These reactions involve compensatory stepping or grasping for support, which are considered swift limb movements [14-19]. In these reactions, age-related declines consisting of shorter step lengths, greater reliance on multi-step responses, increased foot collisions with the stance leg, and lateral deviations after initial steps are well documented [20-23]. These impairments strongly correlate with retrospective and prospective fall risk [23-26]. Hence, to enhance balance confidence and fall resilience, the integration of compensatory stepping interventions within comprehensive prevention frameworks holds promise [27]. Compensatory stepping training (CST) is a targeted perturbation-based intervention that trains these automatic reactive stepping responses through repeated exposure to controlled, unexpected balance perturbations, typically therapist-applied waist pulls or platform translations [27].

The ability to train automatic postural responses is underscored by emerging research. A laboratory study in older adults showed that external perturbations can improve, retain, and generalize compensatory steps. However, for broader applicability, multidirectional training may be needed across slip or trip directions [28,29]. Therapist-applied perturbations or treadmill systems, which are clinically viable methods, are recommended for assessing impacts on fall incidence alongside multi-type and multidirectional protocols, while suggesting scope for long-term trials [28,29].

Therapist-applied postural perturbations require minimal equipment and offer a low-cost and feasible option in diverse clinical contexts, including outpatient rehabilitation clinics, geriatric care settings, and community-based programs. These approaches are particularly suitable for community-dwelling older adults, ranging from relatively healthy individuals to those with frailty, fall history, or neurological conditions such as stroke and Parkinson’s disease [30]. Moreover, because of the higher incidence of lateral instability in older adults, multidirectional training is essential during protective stepping [30]. To reflect real-world scenarios in which falls typically occur, perturbations are recommended to integrate varied sensory-cognitive conditions and functional tasks during dynamic activities such as sit-to-stand transfers or walking in challenging environments, such as obstacles, low lighting, or cognitive distractions. Despite these advances, gaps remain in high-risk older adults regarding the effectiveness of therapist-applied CST related to balance confidence parameters and fall prevention. This scoping review synthesizes the existing literature on the effects of CST during diverse functional tasks and environments, mapping evidence, key concepts, and future research needs.

## Review

The framework of Arksey H and O'Malley L (2005) and the Preferred Reporting Items for Systematic Reviews and Meta-Analyses extension for Scoping Reviews (PRISMA-ScR) [31,32] were followed to frame the present scoping review.

Stage 1: Identification of the research question

The primary research question was, “What is the current evidence on the effectiveness and outcomes of compensatory stepping training compared with conventional therapy for enhancing balance confidence and reducing fall incidence in older adults?” The secondary objectives were as follows: (1) What are the differences in balance confidence between compensatory stepping training and conventional therapy? (2) How do compensatory stepping training and conventional therapy compare in terms of fall prevention and perturbation response metrics? (3) What are the gaps in research regarding multidirectional perturbations, functional task integration, and clinical implementation in diverse settings? (4) What are the recommendations for future research and clinical practice?

Stage 2: Identification of relevant studies

A comprehensive literature search was conducted in the PubMed and ScienceDirect databases for studies published from January 2000 to December 2025. To capture contemporary evidence in older adults relevant to fall prevention practices, the search was limited to English-language studies with free full-text availability. A combination of Medical Subject Headings (MeSH) terms and Boolean operators was used, consisting of (“compensatory stepping”) AND (“older adult” OR “elderly” OR “geriatric”) AND (“fall risk” OR “frail”) AND (“balance confidence” OR “fall prevention” OR “stepping response”).

Stage 3: Selection of studies

The inclusion criteria consisted of studies evaluating compensatory stepping training compared with conventional therapy, including community-dwelling or institutionalized older adults, and reporting outcomes such as balance confidence, fall incidence/rates, perturbation responses, or related measures; studies published in English with full-text availability; and original research articles. Review articles, opinion pieces, non-peer-reviewed articles, editorials, conference abstracts, studies lacking full text or relevance, and studies in non-older populations were excluded. The population, concept, and context framework was as follows: P = older adults aged ≥60 years; C = efficacy of compensatory stepping training versus conventional therapy on outcomes involving balance, fall prevention, and perturbation response; and C = clinical, laboratory, or community settings globally. Titles and abstracts were screened independently by two reviewers, along with the removal of irrelevant studies and duplicates. Full-text articles were then assessed for eligibility, and discrepancies were resolved through discussion.

Stage 4: Data charting

Data were extracted on study characteristics, including authors, year, design, location, sample size, and population; intervention details, including compensatory stepping regimens, perturbation types/directions, duration/frequency, and comparators; and findings on balance confidence, fall prevention, perturbation responses, and feasibility. The identified research gaps and recommendations for future research were also reported. The Mixed Methods Appraisal Tool (MMAT), a widely used instrument for assessing qualitative, quantitative descriptive/cross-sectional, nonrandomized, randomized controlled trial (RCT), and mixed methods studies, was utilised to assess the methodological quality of the included studies. According to the specified criteria, studies were classified as high, moderate, or low quality [33].

Stage 5: Collating, summarizing, and reporting the results

Compared with conventional intervention, an overview of CST efficacy and outcomes was reported, highlighting feasibility, differences in balance confidence, fall prevention, and perturbation responses, along with identified gaps and recommendations for future research. A narrative synthesis, supported by text, tables, and figures, was used to summarize the findings along with thematic analysis.

Results

**Figure 1:**
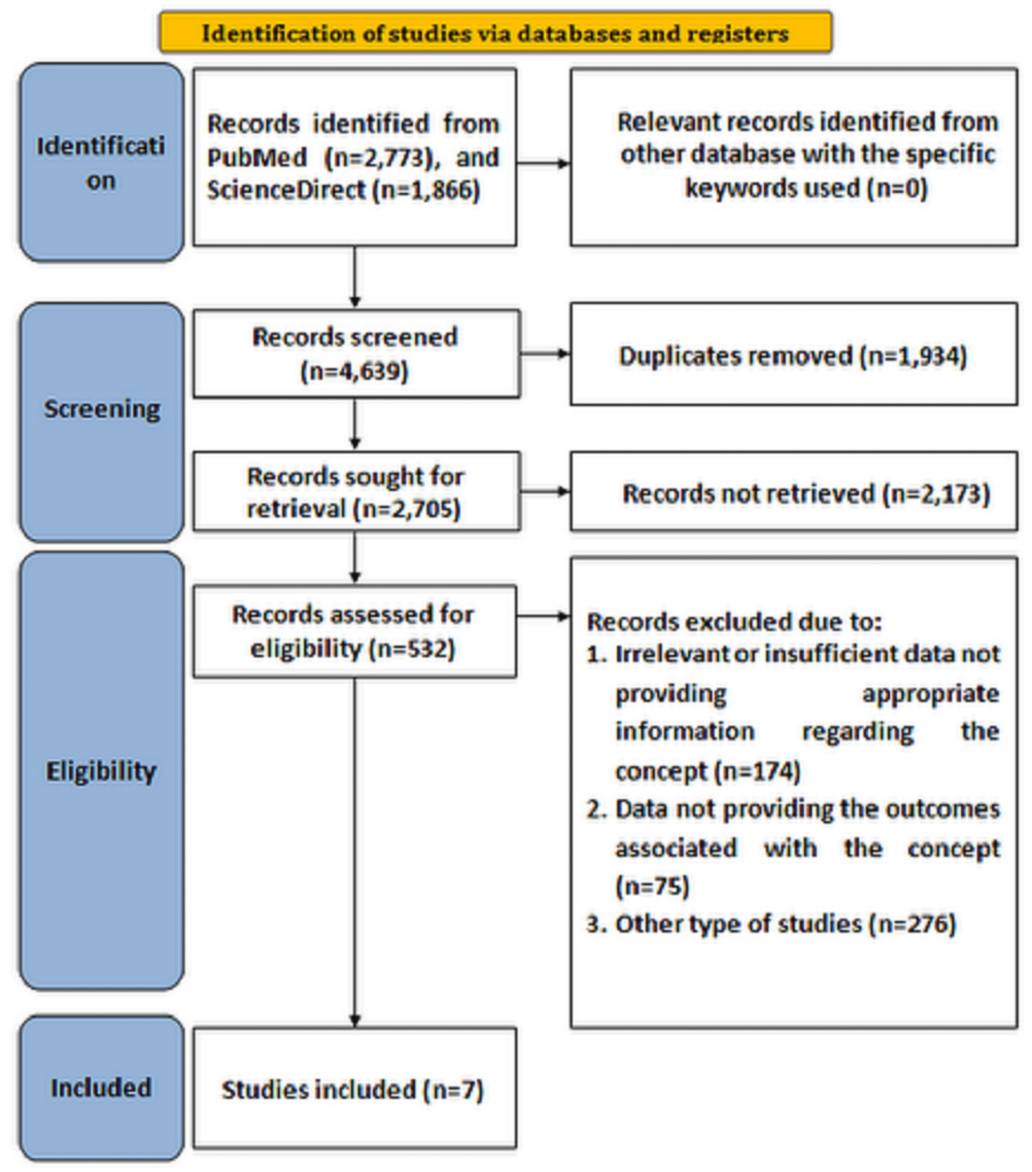
Search strategy and study selection flowchart.

Initially, 4,639 articles were identified after searching the databases from January 2000 to December 2025 using the specified keywords. A total of 1,934 duplicate articles were removed after screening, after which 2,705 articles were sought for retrieval, of which 2,173 articles were not retrieved. Overall, 532 records were assessed for eligibility, of which 525 records were excluded because of irrelevant data not providing appropriate information regarding the concept, data not providing the outcome measures mentioned for evaluation, or other study types. Hence, seven studies fulfilling the eligibility criteria were included in the current review [34-40]. The search strategy according to the PRISMA flowchart is illustrated in Figure 1.

The demographic characteristics of the included studies are reported in Table 1.

**Table 1:** Demographic characteristics of the included studies. RCT: Randomized controlled trial; SSE: Square-stepping exercise; SB: Strength and balance training; IG: Intervention group; CG: Control group.

Author and year	Study design	Study setting	Sample size	Population	Quality
Shigematsu R et al. (2008) [34]	RCT	Mie University, Japan; SSE sessions at a local health center; walking sessions outdoors	68 community-dwelling older adults randomized to SSE (n = 34) or walking (n = 34)	Community-dwelling older adults aged 65-74 years	High
Shigematsu R et al. (2008) [35]	RCT	Kawage Town, Mie Prefecture, Japan	39 community-dwelling older adults randomized to SSE (n = 20) or SB (n = 19)	Healthy, independently living adults aged 65-74 years	High
Fung L et al. (2012) [36]	Three-group non-RCT	Hong Kong, China	31 community-dwelling older adults analyzed: exercise (n = 16) and control (n = 15); original recruitment: exercise (n = 23) and control (n = 20); seven dropouts from the exercise group	Community-dwelling older adults aged 61-86 years	High
Schoene D et al. (2013) [37]	RCT	Independent-living units of a retirement village, Sydney, New South Wales, Australia	37 older adults randomized: IG (n = 19) and CG (n = 18); 32 completed the study: IG (n = 16) and CG (n = 16); five dropouts, with reasons not specified	Community-dwelling older adults aged ≥65 years	High
Skjæret-Maroni N et al. (2016) [38]	Experimental crossover study	Norwegian University of Science and Technology, Trondheim, Norway	20 older adults	Community-dwelling older adults aged 65-90 years	High
Wright RL et al. (2017) [39]	Pilot feasibility study	University of Birmingham and West Midlands Rehabilitation Centre, Birmingham, United Kingdom	12 community-dwelling participants	Community-dwelling participants with a mean age of 60-70 years	High
Rogers MW et al. (2021) [40]	RCT	University of Maryland School of Medicine, Baltimore, Maryland, USA	102 participants randomized: IST (n = 25), HST (n = 25), CMB (n = 25), and SFR (n = 27); 78 included in the final analyses after dropouts: CMB (n = 17), IST (n = 20), HST (n = 19), and SFR (n = 22)	Community-dwelling older adults aged ≥65 years	High

Moreover, the various findings on the effect of compensatory stepping training, outcomes on balance, fall prevention, and perturbation response, along with research gaps and future recommendations from the included studies, are reported in Table 2.

**Table 2:** Summary of the included studies. SSE: Square-stepping exercise; SB: Strength and balance training; TUG: Timed Up and Go test; PPA: Physiological Profile Assessment; CSRT: Choice stepping reaction time; CoM: Center of mass; DGI: Dynamic Gait Index; IST: Induced step training; HST: Hip strengthening training; CMB: Combined training; SFR: Stretching and flexibility relaxation control group; BTL: Balance tolerance limit; RCT: Randomized controlled trial; TOLS: Timed one-leg stance.

Author and year	Intervention details	Balance outcomes	Fall prevention outcomes	Perturbation responses	Feasibility	Research gaps	Recommendations for future research
Shigematsu R et al. (2008) [34]	Duration/frequency: 12 weeks; SSE: 70-minute sessions twice weekly; walking: 60-minute supervised sessions once weekly, plus self-monitored increase in daily steps. SSE regimen: Low-cost indoor square-stepping exercise on a 250 × 100 cm mat divided into 24 squares measuring 40 × 40 cm; participants followed instructor calls for multidirectional stepping patterns, including forward, backward, lateral, oblique, and crossover steps; progressive complexity; no external perturbations, but the intervention emphasized reactive/proactive balance through stepping sequences; warm-up/cool-down included. Perturbation types/directions: None; voluntary stepping patterns, not induced perturbations. Comparator: Walking group involving outdoor supervised brisk walking and pedometer use to increase daily steps.	SSE showed significant group × time interactions favoring improvements in leg power, balance, agility, reaction time, and one self-reported scale. Time effects in both groups included improvements in chair stands, functional reach, and standing from a lying position, with no interaction. Overall, SSE was superior to walking for multiple lower-extremity fitness and balance measures.	At 8-month follow-up, fall rates per person-year were 23.4% in the SSE group versus 33.3% in the walking group (p = 0.31, not statistically significant). This suggests a potential reduction in fall risk factors, but no significant difference in actual falls.	Not directly assessed; no external perturbations were used. SSE indirectly targeted compensatory stepping through patterned, multidirectional voluntary steps on a mat, which may enhance reactive balance responses, but specific measures such as step latency or CoM displacement were not reported.	Adherence/completion was not clearly reported in the available text. No adverse events were reported, and the low-cost indoor SSE format was designed for safety and accessibility in older adults.	Limited statistical power for fall incidence because of a small sample and a non-significant result (p = 0.31). No long-term follow-up beyond 8 months; unclear durability of improvements. The study focused on healthy community-dwelling older adults, with limited evidence in frail individuals, fallers, or clinical populations. Direct perturbation testing was lacking to link stepping patterns to real-world fall recovery. The comparison was mainly with walking; comparisons with other balance/strength programs are needed.	Larger trials are needed to confirm fall-reduction efficacy. Long-term studies should assess retention and real-world generalization. Future studies should test higher-risk groups, such as previous fallers and frail older adults; incorporate perturbation-based assessments to evaluate compensatory stepping improvements; and explore integration of SSE with cognitive or strength elements for multifaceted fall prevention.
Shigematsu R et al. (2008) [35]	Duration/frequency: 12 weeks; 70-minute supervised group sessions twice weekly, including a 15-minute warm-up, 40-minute main exercise, and 15-minute cool-down; participants continued routine activities with no additional exercise. SSE regimen: Performed on a 250 × 100 cm felt mat divided into 40 squares measuring 25 × 25 cm; fast toe-walking following instructor-demonstrated patterns, including forward, backward, lateral, oblique, and crossover steps; 196 patterns in eight progressive difficulty levels; self-selected cadence from slow to fast; no written instructions; emphasized agility and balance through multidirectional voluntary steps. Perturbation types/directions: None; voluntary patterned stepping, not induced perturbations. Comparator: SB training, including 20-minute strength training with three sets of 10 repetitions of squats, lunges, leg extensions, hip abduction/adduction, calf raises, curl-ups, and back extensions using body weight or rubber bands with progressive resistance, plus 20-minute balance training involving one-leg/two-leg stands on toes/heels, tandem stand/walk with eyes open/closed, and seated leg lifts with 10-second holds or 5-m walks.	Significant time effects were observed in both groups in seven of nine functional fitness tests: chair stands, leg extension power, one-leg stand, 5 × 10-m walk, figure-8 walk, 2.44-m Up-and-Go, and 10-m walk (p < 0.05). No group × time interactions were observed, indicating that SSE was not superior to SB. No changes were reported in self-reported scales, including fear of falling, exercise pleasure, and perceived health. SSE indirectly enhanced balance/agility through stepping patterns and was comparable to SB.	At 14-month follow-up, fall incidence was 30.0% in the SSE group, with seven falls in six participants, versus 57.9% in the SB group, with 12 falls in 11 participants; the difference was not significant (p > 0.05). The falls-per-trip rate was 17.1% in the SSE group versus 50.0% in the SB group (p < 0.05), favoring SSE. This suggests that SSE may reduce fall risk more effectively than SB in real-world tripping scenarios.	Not directly assessed; no external perturbations were used. SSE promoted reactive-like stepping through rapid multidirectional patterns, potentially improving fall recovery, but measures such as step latency or CoM displacement were not reported.	Adherence was 100%, with no dropouts. Sessions were supervised by a certified instructor with first-aid training and peer support; participants were encouraged to report symptoms, with none noted. The program was low cost, mat based, group based, and enjoyable, with no adverse events reported; it was suitable for community settings.	The small sample limited power for detecting group differences and fall incidence. No significant fitness superiority of SSE over SB was observed. Long-term retention was not tested, and self-reported outcomes were unchanged, limiting insight into psychological factors. Direct perturbation/fall-recovery assessments were lacking. The study focused on healthy non-fallers rather than high-risk groups. Mechanisms, such as neuromotor adaptations from SSE patterns, remain unclear.	Larger RCTs are needed to confirm SSE’s fall-reduction efficacy and superiority in tripping scenarios. Future studies should include perturbation-based outcomes and higher-risk populations, such as fallers and frail older adults; assess long-term adherence, retention, and psychological benefits; compare SSE with other low-cost programs, such as Tai Chi; and explore dose-response and home-based adaptations for broader implementation.
Fung L et al. (2012) [36]	Duration/frequency: 10 weeks; 28 sessions of 75 minutes each, three times weekly, skipped if there was a public holiday. PSP regimen: Separate sessions for HM/LM groups; 10-minute warm-up with calisthenics to music; 10-minute seated stretching; main exercises included: (a) three-person team relay stepping over a low obstacle on a 6-m course, progressing from 3 to 4 to 6 minutes at sessions 10 and 16; (b) step-on-line exercise using 6-m diverging lines requiring a wide stance, progressing from 3 to 4 to 6 minutes; and (c) grand stepping tour involving a circuit with colored squares, rubber cushions, and low obstacles, performed non-stop and progressing from 6 to 8 to 10 minutes with weekly increases in complexity. Perturbation types/directions: None; voluntary, progressive multidirectional stepping with obstacles for balance challenge, with no external perturbations. Comparator: Control group (n = 15), with no intervention for 3 months; participants were instructed to maintain routine lifestyle and minimal changes, with post-delay TUG retest for eligibility.	Significant group differences in TUG (dynamic balance/mobility; F=11.05, p<0.05, no group effects). PSP is effective for dynamic lower limb function, especially in the lower mobility subgroup.	Not directly assessed; no prospective fall tracking or risk-factor analysis beyond TUG/TOLS was reported.	Not assessed; no external perturbations were used. The program focused on voluntary stepping with progressive obstacles to mimic balance challenges, but reactive measures such as step latency or CoM displacement were not reported.	Seven participants dropped out from the exercise group. Sessions were supervised by consistent instructors and optimized for space/group size. No adverse events were reported. The program was designed to have low cognitive demands, with simple and repetitive patterns.	The nonrandomized design limits causal interpretation, and the small sample reduces power for subgroup analyses. No fall incidence/outcome data were reported. Outcomes were limited to lower-limb function, mainly TUG/TOLS. No long-term follow-up was conducted. Mechanisms, such as strength versus coordination gains, remain unclear. The study focused on community-dwelling older adults and was not tested in frail individuals, fallers, or with perturbations. Static balance, measured using TOLS, did not improve, suggesting program limitations.	Randomized trials with larger samples and long-term follow-up are needed for fall prevention. Future research should incorporate perturbation-based assessments and multiple outcome measures, such as strength and agility; test alternative modes for improving static balance; explore higher-risk groups; and investigate dose-response, cognitive demands, and integration with music/dance elements.
Schoene D et al. (2013) [37]	Duration/frequency: 8 weeks; unsupervised home-based intervention; self-paced, with a recommended frequency of two to three sessions weekly, 15-20 minutes each; plus one supervised CSRT task weekly at the research center. Compensatory stepping regimen: Modified Stepmania, an open-source DDR exergame, using a pressure-sensitive step pad connected to a TV. Participants stepped on directional panels, such as arrows/stars, to match drifting on-screen arrows for timing/direction and returned to central stance panels. Feedback included perfect/good/miss responses and scoring; random game “bombs” were used for response inhibition and cognitive challenge. Participants selected music. Three progressive difficulty levels were used: easy, 72 steps/8 bombs; medium, 80/12; hard, 116/18; with varying speed of 3.2-6.3°/s, steps/sec of 0.5-1, and multiple arrows. Perturbation types/directions: None; voluntary multidirectional stepping to visual cues, with no external perturbations. Comparator: Control group with no intervention/usual activities.	The IG improved PPA composite fall-risk score (F = 12.706, p = 0.001) and subscores, including postural sway (F = 4.226, p = 0.049) and contrast sensitivity (F = 4.415, p = 0.044). No significant changes were observed in other mobility/neuropsychological measures, such as TUG and TUG dual-task, except for verbal fluency/executive function.	Indirect fall-related outcomes were reported. The IG reduced overall physiological fall risk, measured using PPA, compared with the CG; however, prospective fall tracking was not conducted. Findings suggest potential fall-risk reduction through improved stepping/balance parameters.	The IG improved choice stepping reaction time (CSRT; F = 18.203, p < 0.001), a measure of reactive stepping accuracy/speed under choice conditions. Dual-task stepping also improved, reflected by TUG with verbal fluency (F = 4.226, p = 0.049), indicating better cognitive-motor interference handling.	Median adherence in the IG was 2.75 sessions per week. High enjoyment and motivation were supported by game elements, with 86.5% retention. No adverse events were reported. The program was safe for unsupervised use, with no harness used at home, easy setup, minimal technical issues, and suitability for low-risk older adults.	The small pilot sample limits power and generalizability. There was no long-term follow-up or direct fall incidence data. The study focused on low-risk, independent older adults and was untested in frail individuals, fallers, or those with comorbidities. Real-world perturbation testing was lacking. Dose-response and mechanisms, such as cognitive versus physical gains, remain unclear. There was no comparison with other exercises, and adherence beyond 8 weeks is unknown.	Larger RCTs with fall outcomes and long-term retention/adherence tracking are needed. Future studies should test higher-risk groups, such as previous fallers and individuals with cognitive impairment, and integrate supervised components. Multidirectional and real-perturbation generalization and dose effects should be examined. Exergame scalability, cost-effectiveness, and combinations with traditional programs should also be explored.
Skjæret-Maroni N et al. (2016) [38]	Duration/frequency: Single laboratory session; five 1-minute trials per game/level combination, with approximately 20 minutes of total active play. Compensatory stepping regimen: Voluntary multidirectional stepping on a pressure-sensitive mat to match on-screen cues, such as arrows and moles; upper-body movements were encouraged; reflective markers were placed on the toes, heels, and lower back for 3D tracking. Perturbation types/directions: None; cue-based voluntary stepping in ML/AP directions, with no external perturbations. Comparators: Games included The Mole (SilverFit), involving slower and varied steps, and LightRace (YourShape: Fitness Evolved), involving faster and more linear steps; difficulty levels included easy versus medium, with increased speed/complexity.	Game/level affected stepping and upper-body kinematics. The Mole elicited shorter steps, lower velocity, greater step length/velocity variability, larger ML/AP upper-body excursions, and greater area covered by the feet/back compared with LightRace. The medium level reduced overall movement amplitude, except steps/speed in LightRace, suggesting adaptive challenge. These findings indicate that exergames can target balance-relevant functions, such as variability for adaptability and sway for stability.	Indirect fall-related movement characteristics were reported. Improved stepping characteristics, such as variability and speed, are linked to fall-relevant movement patterns, but prospective falls were not assessed. Findings support exergaming for training fall-relevant movements, although game-specific effects imply the need for tailored selection for fall prevention.	Not assessed; no external perturbations were used. The study focused on voluntary stepping responses to game cues, which may mimic reactive balance demands but lack true perturbation dynamics.	Feasibility was high: all 20 participants completed trials without issues. The short duration minimized fatigue, and the motion-capture setup was noninvasive. No adverse events were reported, and games were engaging for older adults based on prior studies.	Limited understanding of exergame-induced movements hinders interpretation of intervention efficacy. The study had a small sample, single-session design, and no assessment of training effects or durability. Only two games/levels were tested, limiting generalizability. There were no direct links to clinical outcomes such as falls or function. The intervention was untested in frail individuals or fallers and lacked perturbation integration for reactive stepping assessment.	Larger studies with multi-session training are needed to evaluate adaptations, retention, and fall outcomes. Future research should test diverse games and populations, such as frail individuals and those with cognitive impairment, with perturbation elements. Dose-response and mechanisms, such as neuromotor changes, should be quantified, and games should be designed for specific balance/fall-prevention goals. Exergames should also be compared with traditional exercises for targeted functions.
Wright RL et al. (2017) [39]	Duration/frequency: Two 3-week blocks, with randomized order; session frequency/duration was unspecified, but the intervention involved home-based stepping in place. Compensatory stepping regimen: Auditory-cued stepping in place to music overlaid with a metronome, using preferred music selected by participants. Tempo increased by 5% weekly for progression, with a focus on rhythmic stepping to improve gait timing, asymmetry, and adaptability. Perturbation types/directions: Phase-shift perturbations, involving random 100-300 ms delays/advances in the metronome beat in one block to cue stepping adjustments; no physical perturbations were used, and perturbations were auditory only. Comparators: Regular metronome block versus phase-shift perturbation block; no inactive control.	Significant improvements from baseline to post-training were maintained at follow-up: walking speed improved from 0.61 to 0.76 m/s, TUG time improved from 20.0 to 16.3 seconds, and DGI score improved from 14.5 to 16.0. No differences were observed between the regular and phase-shift blocks.	Not directly assessed; prospective falls were not tracked. Indirect fall-related improvements were observed through gait speed, functional mobility measured using TUG/DGI, and adaptability, which are fall-risk factors in stroke survivors.	Phase-shift auditory perturbations cued stepping adjustments. No quantitative measures of response accuracy or latency were reported. No differences in outcomes were observed compared with regular cueing, suggesting that both approaches may be effective for timing adaptations.	Enjoyment was high among all participants, and 75% completed both blocks. The home-based setup was feasible with minimal equipment, including a metronome app/music player. No adverse events were reported, and the intervention was well tolerated despite hemiparesis.	The small pilot sample limits power. There was no control group or long-term follow-up beyond the immediate postintervention period. The study was stroke-specific, focusing on chronic hemiparesis, and generalizability to non-stroke older adults or acute phases is unclear. Detailed kinematic/gait asymmetry outcomes were lacking, as were direct perturbation-recovery tests. Unspecified training dose, including sessions per week, limits replication.	Larger RCTs with controls, long-term follow-up, and fall incidence tracking are needed. Future research should incorporate objective gait biomechanics, such as asymmetry measures, and real-world perturbation tests. Dose/frequency should be optimized; diverse stroke subgroups should be tested; and integration with other therapies should be explored. Phase-shift efficacy should also be examined using more challenging and variable perturbations.
Rogers MW et al. (2021) [40]	Duration/frequency: 36 sessions over 12 weeks, three times weekly; each session lasted approximately 60 minutes, with a 10-minute warm-up/cool-down; home maintenance program post-training, two to three times weekly for 3 months. IST: Lateral waist-pull perturbations using a motorized system in right/left directions, with 43 pseudorandom pulls per session at progressive intensities starting 15% above baseline BTL and increasing 10% based on performance; instructions were to react naturally and minimize steps; customized BoS alterations were used if no stepping occurred. HST: Three exercises, each with three sets of 10 repetitions: (1) supine hip abduction with Thera-Bands and progressive resistance; (2) side-lying hip abduction with cuff weights at 35%, 55%, and 75% 1RM; and (3) standing isometric hip abduction against a force transducer with 6-second holds and 5–10 repetitions. CMB: Alternating IST and HST sessions. SFR: Control condition involving seated flexibility/relaxation, including 20 minutes of stretching major muscle groups with 10 repetitions/15-second holds each and no outside practice.	Overall recovery steps were reduced by 31% (main effect p = 0.033). CMB reduced recovery steps from 1.67 to 0.97, with a trend versus control. The fraction of stable single-lateral steps increased, with CMB showing a rate ratio of 2.68 versus SFR (p = 0.004) and IST showing significant improvement (p = 0.003); HST/SFR showed no significant change. No changes were observed in first step length, time, or speed. Four-Square Step Test performance improved with CMB (p = 0.007 vs. SFR), while IST showed marginal improvement (p = 0.082). Other measures, including Berg Balance Scale, Dynamic Gait Index, gait parameters, ABC Scale, and SF-12, showed no significant group effects.	Prospective falls over 12 months post-training were reduced compared with SFR: CMB rate ratio 0.26 (0.07-0.90), indicating a 74% reduction (p = 0.034); IST rate ratio 0.44 (0.18-1.08), indicating a 56% reduction (p = 0.074); and HST rate ratio 0.30 (0.10-0.91), indicating a 70% reduction (p = 0.034), versus SFR at 0.88 falls/person/year. Overall, interventions reduced falls by 56%–74% compared with control.	BTL increased significantly for CMB (p = 0.021) and IST (p = 0.012) compared with SFR; HST showed no change (p = 0.941). Hip abduction torque increased by 25% with HST (p = 0.006 vs. SFR after outlier removal), with no significant changes for IST/CMB.	Per-protocol adherence was defined as completing ≥34/36 sessions within 13 weeks. There were 24 dropouts among 102 participants (24%) because of new medical diagnoses or inability to commit. No safety-related exclusions occurred during training. No adverse events were explicitly reported. Training was supervised by a physical therapist/research assistant, and minimal safety risk was confirmed before enrollment. Home maintenance compliance was assessed using journals and weekly calls, but was not quantified in the available extract.	The study focused on lateral rather than sagittal perturbations despite older adults’ vulnerability to ML imbalance. Effects of strength training alone on falls were inconsistent, highlighting the need for task-specific integration with balance perturbations. Durability of effects beyond 3 months was not fully addressed here. ML stepping patterns, such as crossovers and collisions, and their links to abductor/adductor muscle changes in fallers remain understudied. Generalizability to frailer populations or real-world perturbations, such as uneven terrain or crowds, is limited.	Lateral perturbation training should be incorporated into traditional fall-prevention programs for enhanced efficacy. Future studies should examine long-term durability beyond 3 months and dose-response of combined versus isolated interventions. Biomechanical and neuromotor mechanisms, such as sensorimotor adaptations, should be investigated in diverse older adult subgroups. Studies should test high-risk groups, such as fallers and frail individuals, in real-world settings, and compare ML versus AP perturbations and multidirectional protocols.

Efficacy in enhancing balance and perturbation responses

Across the studies, CST encompassing perturbation-induced, voluntary patterned, auditory-cued, and exergame-based stepping demonstrated consistent improvements in dynamic balance and reactive stepping metrics, outperforming or complementing conventional therapies, such as strength/balance exercises, in task-specific domains. For instance, Rogers MW et al. reported a 31% reduction in recovery steps post-CST, with combined perturbation-strength training (CMB) yielding a 2.68-fold increase in stable single-lateral steps compared with controls (p = 0.004), alongside enhanced Balance Tolerance Limits (BTL; p = 0.012-0.021) [40].

Balance adaptability was also targeted by voluntary and cue-based stepping variants. Square-stepping exercise (SSE) improved agility and reaction time (p < 0.05), with significant time effects reported by Shigematsu R et al. and Shigematsu R et al. compared with strength-balance training in functional tests such as the Figure-8 Walk and Up-and-Go [34,35]. For lower-mobility subgroups using progressive obstacle stepping, Fung L and Lam M noted a 21.5% gain in Timed Up-and-Go (TUG) scores, though static balance, measured using the timed one-leg stance (TOLS), remained unchanged [36]. Multidirectional efficacy was further emphasized through exergaming approaches, with improvements in choice stepping reaction time (CSRT; F = 18.203, p < 0.001) and dual-task mobility (p = 0.049) reported in a study by Schoene D et al. [37]. Game-specific kinematics were highlighted by Skjæret-Maroni N et al., with “The Mole” eliciting greater step variability and upper-body excursions for adaptability training [38].

Wright RL et al. extended this to timing and rhythm: induced waist-pull training reduced voluntary step initiation by 15-17%, while auditory cues in stroke survivors enhanced gait speed from 0.61 to 0.76 m/s and Dynamic Gait Index (DGI) scores from 14.5 to 16.0 [39]. Collectively, these findings show that conventional therapies demonstrate limited transfer, such as no BTL gains in strength-only groups, whereas CST excels in reactive, multiplanar balance, including lateral/ML perturbations.

Contributions to fall prevention and confidence

Although direct balance confidence measures, such as the Activities-specific Balance Confidence (ABC) Scale, were underreported, CST led to reductions in prospective falls. Compared with controls, Rogers MW et al. provided the strongest evidence, with 56-74% fall rate reductions over 12 months, attributed to fewer multi-step recoveries and collisions [40]. Shigematsu R reported non-significant reductions in fall incidence (23.4% vs. 33.3%; p = 0.31), but SSE lowered falls-per-trip rates (17.1% vs. 50.0%; p < 0.05), emphasizing tripping resilience [34]. Exergame and cue-based studies improved functional mobility, including TUG and DGI, which are key fall predictors, and Physiological Profile Assessment (PPA) scores (F = 12.706, p = 0.001), but lacked prospective tracking. No ABC changes were reported by Rogers MW et al. [40]. This highlights the preventive potential of CST through kinematic efficiency, while also emphasizing a gap in psychological outcomes [34,35].

Feasibility and implementation challenges

Supporting the scalability of CST in diverse settings, no adverse events and high adherence rates of 75-100% were reported across studies. Schoene D et al. reported 86.5% retention, achieved through unsupervised exergaming performed for 2.75 sessions per week [37]. Additionally, Wright RL et al. reported 75% completion of auditory-cued blocks, with universal enjoyment [39]. Supervised clinic or laboratory protocols maintained safety, with per-protocol adherence ≥94% in 12-week programs [40]. Accessibility was enhanced by low-cost elements [34,35,39], although dropouts occurred because of medical conditions [36,40]. Skjæret-Maroni N et al. noted adaptive challenges at higher levels but observed game-specific engagement [38]. Overall, the minimal risk and motivational features of CST, such as music and gamification, position it as feasible for high-risk groups, contrasting with the higher supervision needs of conventional therapies.

Evidence gaps and future directions

Underrepresentation of frail/institutionalized subgroups and confidence measures was observed, with a stroke-specific focus restricting applicability to older adults [39]. Hybrid protocols combining CST with strength or cognitive factors; inclusion of high-risk cohorts with psychological endpoints; implementation trials in diverse settings to evaluate cost-effectiveness; and RCTs with larger samples integrating multidirectional or real-world perturbations for dose-response and durability are recommended for future research.

Discussion

This review maps evidence from seven studies focusing on CST interventions by highlighting their efficacy in enhancing balance, perturbation responses, and confidence. CST showed consistent enhancements in reactive balance metrics, including reduced recovery steps, greater kinematic variability for adaptability, and improved step initiation timing [34,38,40], with no adverse events and high feasibility. Although minimal changes were observed in balance confidence, fall prevention benefits were promising, along with lower falls-per-trip ratios [34,35,40,41]. However, SSE reported equal benefits compared with strength training, and anteroposterior gains were not transferred laterally or to functional outcomes, limiting generalization [34-36]. Moreover, home-based variants highlighted scalability [37,39]; however, gaps related to effectiveness in high-risk frail cohorts and long-term retention were observed.

The mechanism involves neuromotor adaptations that recalibrate central pattern generators for stepping, similar to gait control circuits [40]. Induced perturbations evoke high-intensity afferent feedback [40], facilitating spinal and supraspinal plasticity to reduce multi-step reliance and shorten step latencies, as evident in fewer recovery steps post-training. Similarly, induced waist-pull training outperformed voluntary practice in auditory transfer tasks, as reported by Wright RL et al., suggesting that automaticity is enhanced by sensory-driven entrainment [39]. In contrast, comparable agility gains were observed from voluntary SSE without perturbations, implying that patterned repetition alone may suffice for lower-extremity power but may undertrain reflexive thresholds, as static balance, measured using TOLS, remained unchanged [34-36]. These adaptations may originate from Hebbian-like strengthening of corticospinal pathways, optimizing real-time error correction during dynamic perturbations.

Another key mechanism is sensorimotor integration, as CST refines proprioceptive and vestibular processing for multiplanar stability [28,38]. Through hip torque gains, lateral perturbations boosted BTLs, promoting single-lateral steps over error-prone crossovers, a vulnerability in older adults, as reported by Rogers MW et al. [40]. Exergames reinforced this by eliciting step variability and greater mediolateral (ML) excursions [38], supporting proprioceptive recalibration for environmental adaptability. However, contrasting single-session designs with multi-week protocols highlights dose dependency [37,38]. Although CSRT improved significantly, generalization faltered without multidirectional cues, as backward adaptations were retained but lateral adaptations were not [28]. This underscores the role of afferent-efferent coupling, whereby repeated exposure enhances predictive feed-forward control and minimizes foot collisions.

The transfer of CST to falls is explained by cognitive-motor dual-tasking mechanisms, integrating executive function with stepping under divided attention. DGI and gait speed were enhanced by auditory-cued training [39], reducing asymmetry in stroke survivors through rhythmic entrainment that buffers cognitive load. Schoene D et al. corroborated this with dual-task TUG improvements, attributing gains to inhibitory control from game “bombs” fostering response selection [37,42]. In contrast, simpler SSE patterns reported reductions in fear of falling; however, the results were non-significant, suggesting that cognitive demands must escalate progressively [34,35]. Dynamic TUG improved through obstacle stepping rather than static balance, as stated by Fung and Lam, implying limited prefrontal engagement without perturbations [36]. Collectively, these studies indicate that CST augments frontoparietal networks for interference resistance; however, differences across populations reveal that baseline deficits may predict the magnitude of adaptation, favoring high-risk tailoring.

For high-risk older adults, CST serves as an adjunct to conventional interventions, as it requires minimal equipment, uses therapist-applied perturbations, is low cost, and is feasible in community or home settings. Lab-to-life gaps can be bridged by integrating multidirectional tasks, such as walking with distractions during functional activities, thereby enhancing adoption in telerehabilitation or geriatric clinics. Reductions in healthcare costs and increased scalability through fall prevention may occur by using home-based exergames and cues [40]. However, routine integration of ABC is required for balance confidence, as it was underreported. Combining CST with conventional treatments should be considered, empowering therapists to target vulnerabilities such as lateral instability for improved outcomes.

Limitations

Due to the scoping nature of the review, a meta-analytic evaluation of effect sizes was not feasible. Considerable heterogeneity was observed in populations, interventions, and designs, thereby complicating direct comparisons and synthesis. Moreover, sample sizes were generally small. Evaluation of intervention retention and long-term effectiveness was limited. Infrequent reporting of balance confidence measures was also observed. Additionally, because only English-language literature with full-text availability was incorporated, selection bias may have occurred.

Recommendations for future research

Large-scale, multicenter RCTs are required to evaluate multidirectional CST protocols integrated with environmental challenges and functional tasks. To assess sustained fall reductions and improvements in balance confidence, long-term follow-ups, prospective incidence tracking, and incorporation of validated tools such as the ABC Scale are essential. To optimize protocols for diverse settings, hybrid interventions combining CST with conventional treatments are required. Moreover, to enhance applicability, the inclusion of high-risk subgroups, such as frail individuals, multiple fallers, or those with comorbidities, is necessary. Furthermore, for scalability, evaluations of cost-effectiveness, therapist training, and telerehabilitation adaptations should be implemented. Systematic reviews with meta-analyses could also quantify mechanisms and guide policy for global fall prevention.

## Conclusions

This scoping review illuminates the transformative potential of CST in older adults for mitigating fall risk and strengthening reactive balance as a task-specific, feasible intervention. CST may surpass some limitations of conventional exercises by targeting real-world perturbations through multidirectional, functional protocols. Considering parameters such as adaptability and step efficiency, consistent neuromotor gains were observed across diverse modalities, from perturbation-induced waist pulls associated with fall reductions to home-based exergames enhancing dual-task agility. For fall prevention in older adults, and to empower clinicians to implement scalable, low-cost approaches, CST emerges as an important intervention, not merely an adjunct, that may help restore resilience and autonomy. Future trials incorporating CST may support fall prevention and healthy aging as the aging demographic rises.
